# Miscellaneous

**Published:** 1866-02

**Authors:** 


### MISCELLANEOUS.

*The Cattle Plague; Homœopathic Trial in England.*—In the *Mark Lane Express* of November 27th, we find the following account of the trial of Homœopathy in Norfolk, England, by which it appears this supposed cure has failed:

"At the meeting of the Norfolk Cattle Plague Association, on Saturday, Mr. Forrester made a lengthened statement as to the results which had attended the homœopathic treatment attempted by Mr. Moore. There was a difference of opinion as to the animals proposed to be placed on the trial register, but Mr. Moore's objections were, at the utmost, against three animals only. Mr. Moore wished to have several animals put on the register, which Messrs. Smith and Wells objected to as not being sufficiently developed cases, but inasmuch as these animals were not thereby deprived of homœopathic treatment, the question of their recovery or death was not affected by the course adopted. It was to be regretted that Mr. Allen, in the exercise of his discretion, had thought proper to kill his two cows which were placed on the register; but, after all, it did not affect the question at issue; the cows had been for a long time under treatment by chlorate of potash. Three other cows which were ill at Mr. Allen's were killed on the 15th and 17th, and five more had been killed since, one of which had been under arsenicum for a month. The others had also been killed. All Mr. Allen's cows had been under arsenicum. Of Mr. Reid's cattle, two cows and two bulls were put on the register on the 16th; one cow died on the 20th, and another on the 21st; one bull on the 18th, and the other on the 20th. Mr. Reid's stock had been 41 in all; one was killed, 38 had died, and two buds (those beginning to shows their horns) were now convalescent. At M. C. Atkin's one cow was put on the register; the bull was thought to be sickening, but was not registered; both had been under homœopathic treatment by Mr. Buckenham, the inspector; the cow died on the 22d, the bull on the 23d. Nothing remained on the farm on the previous day (November 24th) but ten grazing bullocks, one of which showed decided symptoms of rinderpest. At Mr. Carman's of Weston, one cow was put on the register on the 16th, and died on the 20th; another cow which was ill on the 16th, died on the evening of that day; of three other cows alive on the 16th, one died on the 23d, another was very ill on the 24th, and the other was convalescent, but the pulse was 84, and the respiration 32. Of ten young beasts two had died, and two were very ill. The stock on this farm had been tended carefully, and had been put under homœopathic treatment as they fell ill. At Mr. Savory's, Sparham, five bullocks, one bull, and three calves had died previous to Monday, November 20; seven others had died since; one had recovered; all were treated with homœopathic medicine. At Mr. English's, of Lynne, two of three cows placed under Mr. Moore's treatment had died. Two buds had died, and two spotted buds were now falling ill, and three calves and two cows were well. A letter was read from Mr. Moore, refusing to proceed further with the trials, on the ground that he had not had fair treatment. Mr. Forrester expressed his opinion that the treatment of animals after they had passed the first stage of the disease had resulted in failure. Lord Bury argued that the result was not conclusive, as Mr. Moore had only been a few hours in Norfolk. A London committee were ready to send down a staff of homœopathic surgeons to work the matter properly, but $1,000 would be required for the purpose. Would the association find a portion of this sum? The matter was, after some discussion, referred to the sub-committee, and the meeting soon afterwards adjourned."

---

*Dr. Kidd's Homœopathy.*—Our faithful and orthodox readers may, perhaps, think us wavering; but we cannot forbear the expression of our satisfaction at the progress of homœopathy, and of a feeling that we should scarcely be sorry if all the cows in the country belonged to Dr. Kidd. Dr. Kidd, it would seem, has cows; and one of these caught the plague by grazing in a field adjoining a meadow where several cows had died a month before. The treatment of this animal by Dr. Kidd afforded the Doctor an opportunity of a letter to *The Times*. One case, it must be admitted, can supply only an infinitesimal amount of experience; but we must take the Doctor's letter for what it is worth, and get as much light from it as we can on the nature of those measures on which homœopathists rely. Homœopathy, according to Hahnemann, was a mystery only intelligible to mystics. In a homœopathic maxim, which he said could "*not be refuted by any experience in the world*," he averred that "the best dose of medicines was always *the very smallest one in the one of the high dynamizations*." His favorite quantity, in his earlier days, was the decillionth of a grain —a quantity which algebraists declare to be unprocurable, and which would require a mass of material for admixture larger than the earth itself. He lived, however, to think this dose too large, and to declare his decided preference for the mere smell of a drug, even if it were destitute of odor. "Olfaction" became his favorite remedy in both chronic and acute diseases. "I can scarcely name," he says, "one in a hundred out of the many patients who have sought the advice of myself and assistant during the past year whose chronic and acute diseases we have not treated with the most happy results *solely by means of this olfaction.*"

Such was Hahnemann's homœopathy, which has always been unintelligible to the uninitiated. Let us compare it with Dr. Kidd's, and our readers will then understand what we mean by the progress of homœopathy, and by our satisfaction at this progress.

Dr. Kidd's cow that had the plague got the following things in the course of its treatment, each of which, for clearness' sake, we shall give separately, and much in the order in which they occur in the Doctor's own narrative:

1. One-tenth of a grain of arsenic every two hours, day and night, finally doubled, making one-fifth of a grain; by no means an impalpable dose that would escape the analysis of Dr. Taylor, as the medicine of the old globules did, seeing that our ordinary allopathic dose is one-sixtieth of a grain.

2. When the arsenic was doubled in quantity, the mysterious principle of alternation was had recourse to, and one-fiftieth of a grain of phosphorus added to the medicine.

3. The warmest and best-ventilated shed.

4. "Quarts of barley-meal gruel poured down" day and night.

5. Filled the shed with steam, and by the labor of four men converted it into a vapor bath.

6. The cow having calved, and thought to be dying, Dr. Kidd got them (the four men) "to pour down her throat four bottles of Barclay's stout in eight hours."

7. The cow being nearly dead, "but determined not to give her up, I ordered the *gruel to be made with old ale*, the bottled stout being also continued."

Little by little she recovered. Dr. Kidd was rewarded for his perseverance by seeing his cow eat hay and take branmashes —by seeing the milk come, and the calf (for it was born alive) take the milk and thrive on it: and, for aught we know, there has been no interruption in the convalescence.

And shall we not rejoice with Dr. Kidd over his recovered cow? We shall and do. We are concerned, indeed, for the peace of Hahnemann. Could he but know that one of his most distinguished disciples had such vulgar notions of "high dynamization" as is represented in the pouring down by four men of these quarts of gruel, Barclay's stout, and old ale—in warm fresh air—and last, and evidently least, in arsenic and phosphorus (not in your imponderable quantities, such as would be got in the smell of a lucifer match, but in highly appreciable and very heavy doses,)—doubtless he might fear that "experience had refuted" his most cherished maxims. But, barring this consideration, how great is the progress of homœopathy! how it rises to sense and science! We could wish, indeed, that Dr. Kidd had told us his confidential opinion as to the respective credit which these various measures are to have in regard to the result—whether the fifth of a grain of arsenic and the fiftieth of phosphorus, which we shall put in one scale, or the air, warmth, gruel, stout, and ale, which we shall put in another, had most to do with the cure. It is due to the Doctor to say that the allusion to medicines is mild and modest. The medicines subside beautifully in the narrative, and disappear in a scene of warmth and comfort and good cheer. But we seek information: which of these things was homœopathic to the disease? Would that all the poor cows had Dr. Kidd and his four men to attend them! We have heard of sad failures in the homœopathic treatment of cattle plague. Which homœopathy was it, may we ask? Was it Hahnemann's or Dr. Kidd's—homœopathy, or allopathy in disguise?—*London Lancet*, February, 1866.

---

*May 3d.* Dr. PACKARD made a verbal communication on the subject of Anæsthesia, as follows:

My attention was attracted a few days since by an article, from the pen of Dr. Lente, of Cold Spring, N. Y., published in the last number of the *New York Medical Journal*, and entitled "Sulphuric Ether *versus* Chloroform." In that article Dr. L., who is well known as an advocate of the use of ether by his writings in various periodicals, says: "This fact I regard as settled—that a patient may be brought under the influence of sulphuric ether as quickly as he can safely by chloroform, and with a quantity costing less, and weighing but little more than the requisite amount of the latter; the objection, then, sometimes raised by army surgeons, of increased trouble of transportation, is not tenable.

"If any doubt this fact, after referring to the statistics above alluded to,* *I will agree to go to any hospital where a large number of operations are being performed, and demonstrate it to the satisfaction of the opponents of ether.* * * * *

---

* Given by Dr. Lente in the American Journal for April, 1861, in the American Medical Times, June 28, 1862, and in a Report of the Committee of the Boston Society for Medical Improvement, published in 1861.

"The recently published report of the Royal Medical and Chirurgical Society of London, adverse to the employment of sulphuric ether, and the recent occurrence of so many additional cases of death from chloroform poisoning, have forcibly recalled my attention to this subject, and at the risk of charge of egotism, induced me to make the offer which I here repeat; in other words, that is, to guarantee to get a number of patients, in any hospital, under full anæsthesia with sulphuric ether in a short a time as can safely be done with chloroform, and with a quantity not exceeding an average of two ounces and a half, the average time two minutes and a half. The average time and quantity would probably be less in the ordinary run of hospital cases (not more than half as much in cases of considerable debility, and after hemorrhage or insufficient nourishment, as in many cases in military surgery.")

Until the fall of 1864, I had been in the constant habit of administering chloroform for the purpose of inducing anæsthesia, and in hundreds of cases have never seen any injurious effect from it. My reason for preferring it to ether was the greater facility of carrying it, the very much smaller quantity required, and the more agreeable character of its influence. A case which occurred then, however, (reported by me to the College, and published in the *American Journal* for January, 1865,) in which death was imminent, and averted only by the prompt use of an electro-magnetic battery, shook my confidence; and it was shortly followed by another, in which death actually took place, at the Beverly U. S. A. Hospital.

Another case has very recently occurred to me, in private practice, in which chloroform, given to a patient already nearly insensible from ether, was productive of alarming symptoms.

Dr. Lente's statements, as before quoted, led me to write to him to inquire as to the method of administering the ether, in order to obtain the effect in so short a time, and with so small a quantity of the article. His answer to my question is contained in the following extract from his letter:

"All you want for the efficient administration of ether, after procuring a pure article (and Squibb's I prefer to any other,) are stiff paper and a rather stiff and thick towel, if one can be had—if not, any will do. I usually take a newspaper—as that is to be had in every house—fold it, so as to make it about eighteen inches long and seven to eight broad; fold the towel so as to correspond, lay one on the other, fold them so as to form a cone, with the *towel inside*, and pin them securely, especially the edges of the towel inside, so that it will not fall on the face and annoy the patient. The apex of the cone must be folded tightly, so that *no air* can enter. If the cone is rather elongated, I stuff a white handkerchief tightly into it, so not to have its capacity too great. You see the great object is to have the vapor *as concentrated as possible*, just the reverse of what is safe with the vapor of chloroform. The patient being all ready, I explain to him fully how to inhale the ether, and the unpleasant symptoms which he will probably experience at first, assuring him of the perfect safety of the process (which cannot be done in the case of chloroform.) I then have some one to take hold of his hands quietly, so as to be ready to arrest any sudden movement towards tearing away the inhaler from the face, and other assistants to look out for other violent movements, so that the process, when once commenced, shall not be interrupted for a moment. I then pour on, from a three or four ounce bottle, with not too narrow a mouth, about a drachm or two of ether (if I am anxious to use as little as possible,) if not a little more, until I ascertain the capacity of the patient for breathing it; if he does not cough or strangle, I put it in close contact, taking care always not to press in the sides of the cone, so as to encroach on its capacity, holding it with *both hands*, near its edges, and pressing them pretty firmly, at all points, against the face. If he persistently hold his breath, as patients occasionally do, or strangle in any considerable degree, I remove it *slightly* from the face for a moment. In a very short time, I throw on an ounce more of ether, and then keep the inhaler in close contact with the face; this is repeated, scarcely ever using more than three or four drachms at each fresh application, until the etherization is effected. A very important point, and one most generally neglected, is to keep the inhaler away as short a time as possible when replenishing the ether, *throwing it on*, and not deliberately pouring it on, as I usually see done. As long as the patient retains consciousness, if he does not follow my directions with regard to full and rapid inspirations, I now and then call loudly, in his ear, *breathe strongly!* No matter how hard his struggles are, after he has commenced to breathe fully, I never 'let up,' but keep the cone remorselessly pressed against his face, and ply him still more strongly with the ether. This must be especially attended to in the case of all children, for with them it is generally a struggle from the beginning, whether ether or chloroform be used, and it is neither necessary nor practicable to get their confidence so as to ease them with the inhalation, as I have described above in the case of adults. Their cries and consequent full inspirations cause them to succumb very rapidly.

"By following these directions you will, after a little practice, etherize your patients as quickly and with as little trouble as you can safely chloroformize them—that is, in from three to four minutes (average,) and with from one and a half to two and a half ounces of ether. But if you choose to use from *two to three* ounces of the drug—and more than the latter is almost never necessary—you may shorten the time by a minute. I speak of comparatively robust adult subjects; with feebler, less time and less ether are required. It is better to have no current of air about the patient during the process, if possible. It is important to be fully impressed with the fact that there is absolutely no danger of death from *too sudden action* of the ether, otherwise we will not give it with sufficient confidence to insure a prompt result. The little danger attaching to ether is from *prostration*, which always occurs after the operation is over, and the patient has recovered his consciousness. I have, in several communications, insisted that the pulse should be watched for a time after the administration of ether, especially in delicate subjects, or if there are any indications of extreme debility. I have had, and have published several cases of severe prostration succeeding etherization, but I have never seen any case where I could get a patient too suddenly under the effect of sulphuric ether. It is not surprising that when the manner of administering the two agents is so entirely different, those who have generally been in the habit of using one, should fail when attempting to employ the other, until they have become thoroughly accustomed to it. I gave ether a short time ago to myself, in the hospital at West Point, unassisted, and became completely insensible with half an ounce, so as to be unconscious of the extraction of a molar tooth by the hospital steward, Mr. Saunders. A few days after, with a four ounce bottle of ether, I etherized three patients, from whose jaws the steward extracted at least forty teeth in the aggregate, and used only about three-fourths of the contents of the bottle. As regards the *nausea* and *vomiting*, referred to in your letter, if no food be taken for four or six hours before the operation, there is seldom any trouble worth mentioning. It is usually well to give a little brandy and water or a glass of wine before the operation, especially if the subject be feeble. And in case of severe operations, if much prostration supervenes, I give an enema of brandy and water. I have thought for some time of having an inhaler constructed of silver wire network, with a cone of woolen over it, and some impervious material over that, as suggested by my friend, Prof. T. G. Thomas, but I succeed so well with the extemporized apparatus above described, that I have not yet had it done."

I have not yet had an opportunity of trying this method of administering ether, but, from what I know of Dr. Lente, I am inclined to place great confidence in what he says. It is very doubtful whether we ever obtain any other anæsthetic agent at once so safe and so efficient as sulphuric ether; we certainly have not found any such as yet. If, therefore, we can improve upon the methods now generally in vogue for its administration, we shall do well to prefer it to its more dangerous rivals.—*Am. Jour. of the Med. Sciences*, Jan., 1866.

---

*Modification in Canquoin's Caustic Paste.*—This valuable caustic would be still more employed, were its application not somewhat difficult; and one of M. Demarquay's pupils has contrived a modification in its composition which renders its application very easy and effectual. The paste thus formed consists of chloride of zinc ten, flour twenty, and glycerine four parts. So prepared, it can be applied to the part to be destroyed with great facility, however varied this may be in shape or direction, and can easily be washed away. M. Demarquay has frequently employed it, and finds the paste thus prepared with glycerine instead of water far preferable, both with respect to its application and the results.—*Med. Times and Gaz.*, Dec. 2, 1865, from *Bull. de Therap.*, Sept. 15.

